# Tetraspanin CD151 as an emerging potential poor prognostic factor across solid tumors: a systematic review and meta-analysis

**DOI:** 10.18632/oncotarget.13532

**Published:** 2016-11-23

**Authors:** Ping Zeng, Yin-Hua Wang, Meng Si, Jin-Hua Gu, Ping Li, Pei-Hua Lu, Min-Bin Chen

**Affiliations:** ^1^ Department of Radiotherapy and Oncology, Kunshan First People's Hospital Affiliated to Jiangsu University, Kunshan 215300, Jiangsu Province, China; ^2^ Department of Oncology, Changshu Second People's Hospital Affiliated to Yangzhou University, Changshu 215500, Jiangsu Province, China; ^3^ Department of Neurology, the Second Affiliated Hospital of Soochow University, Suzhou 215004, Jiangsu Province, China; ^4^ Department of Medical Oncology, Wuxi People's Hospital of Nanjing Medical University, Wuxi 214023, Jiangsu Province, China

**Keywords:** CD151, solid tumors, prognosis, overall survival, disease-free survival

## Abstract

Tetraspanin CD151, also known as PETA-3 or SFA-1, has been reported to predict prognosis in various solid tumors. Yet, the results of these studies remained inconclusive. Here, we performed this meta-analysis of relevant studies published on the topic to quantitatively evaluate the clinicopathological significance of CD151 in solid tumors. The relevant articles were identified via searching the PubMed, Web of Science and Embase database. The pooled hazard ratios (HRs) and corresponding 95% confidence intervals (CI) of overall survival (OS) and disease-free survival (DFS) were calculated to evaluate the prognostic value of CD151 expression in patients with solid tumors. A total of 19 studies involving 4, 270 participants were included in the study, we drew the conclusion that CD151 overexpression was associated with statistically significant poor OS (pooled HR = 1.498, 95% CI = 1.346-1.667, *P*<0.001) and poor DFS (pooled HR = 1.488, 95% CI = 1.314-1.685, *P*<0.001). Furthermore, the subgroup analysis revealed that the associations between CD151 overexpression and the outcome endpoints (OS or TTP) were significant within the Asian region and European, as well in patients with breast cancer or gastric cancer. Taken together, the incorporative HR showed CD151 overexpression was associated with poor survival in human solid tumors. CD151 could be a valuable prognosis biomarker or a potential therapeutic target of solid tumors.

## INTRODUCTION

Tetraspanins CD151, a known transmembrane 4 superfamily protein, contains several different structure domains. Expression of CD151 was observed in different cell types [1]. CD151 and other tetraspanins participate in a variety of key cellular functions [1–3]. CD151 is the first characterized member of the tetraspanin family. It is also known as glycoprotein-27 (GP-27), Red blood cell antigen MER 2 (MER 2), platelet-endothelial tetraspan antigen-3 (PETA-3), Raph blood group antigen (RAPH)/Membrane Glycoprotein SFA-1 (SFA-1) and tetraspanin-24 (TSPAN-24), was located on chromosome 11p15.5 [4, 5]. Its expression was found in a number of cell types [6]. Altered expression of CD151 was involved in cancer growth [7], progression [8], motility, invasion, and metastasis [9]. Existing evidences have demonstrated that CD151 overexpression was observed in several malignancies [10–23]. These striking evidences on the role of CD151 in cancer suggest the tetraspanin would be further considered as a novel potential oncotarget of cancer patients.

An increasing number of studies showed that increased CD151 expression in tumor tissue was associated with cancer patients’ poor survival [10–13, 15, 17–27]. However, in invasive lobular breast cancer [15] and endometrial cancer [16], overexpression of CD151 might have diverse, even opposing roles, correlating with better clinical outcomes. The results of those individual studies were inconsistent. Thus, this comprehensive meta-analysis was performed to clarify the possible prognostic role of CD151 expression in solid tumors.

## RESULTS

### Demographic characteristics

**Figure 1:**
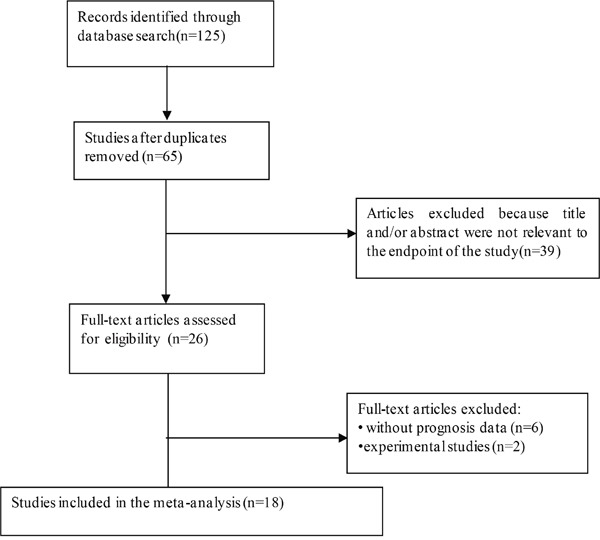
The flow chart of the selection process in our meta-analysis

125 articles were retrieved from the different databases (PubMed, Embase, and Web of Science). As showed in the search flow diagram (Figure 1), 125 records were initially retrieved through the pre-defined search strategy. Because of repeated data, 65 records were removed. After browsing the retrieved titles and abstracts, 39 records were excluded due to no relevant endpoint provided. The remaining 26 records were downloaded as full-text and accessed very carefully. Among them, another 18 studies were excluded, including 1 study that was experimental study, 7 studies that were without prognosis data. After selection, 18 published studies including 4, 270 patients were finally selected for this meta-analysis. The median sample-size was 145, with a wide range from 30 to 886. Among all cohorts, Asian (n = 14) became the major source region of literatures, followed by Austria (n = 1), Poland (n = 2), USA(n = 1), UK (n = 1). Newcastle-Ottawa Scale (NOS) were applied to analyze these studies. The quality scores of these studies ranged from 6 to 9, indicating that the methodological quality was high.

As for the cancer type, two studies evaluated non-small cell lung cancer; Two studies focused on glioblastoma; Three studies evaluated breast cancer; One study evaluated gallbladder carcinoma; Three studies evaluated gastric carcinomas; One study evaluated CCRCC or clear cell renal cell carcinoma; One study evaluated esophageal squamous cell carcinoma; One study evaluated pancreatic cancer; Two studies evaluated liver cancer; One study evaluated prostate cancer; One study evaluated colon cancer, and one study evaluated endometrial cancer. Of all the studies, 19 of them focused on OS, other 10 studies focused on DFS.

### Evidence synthesis

**Figure 2:**
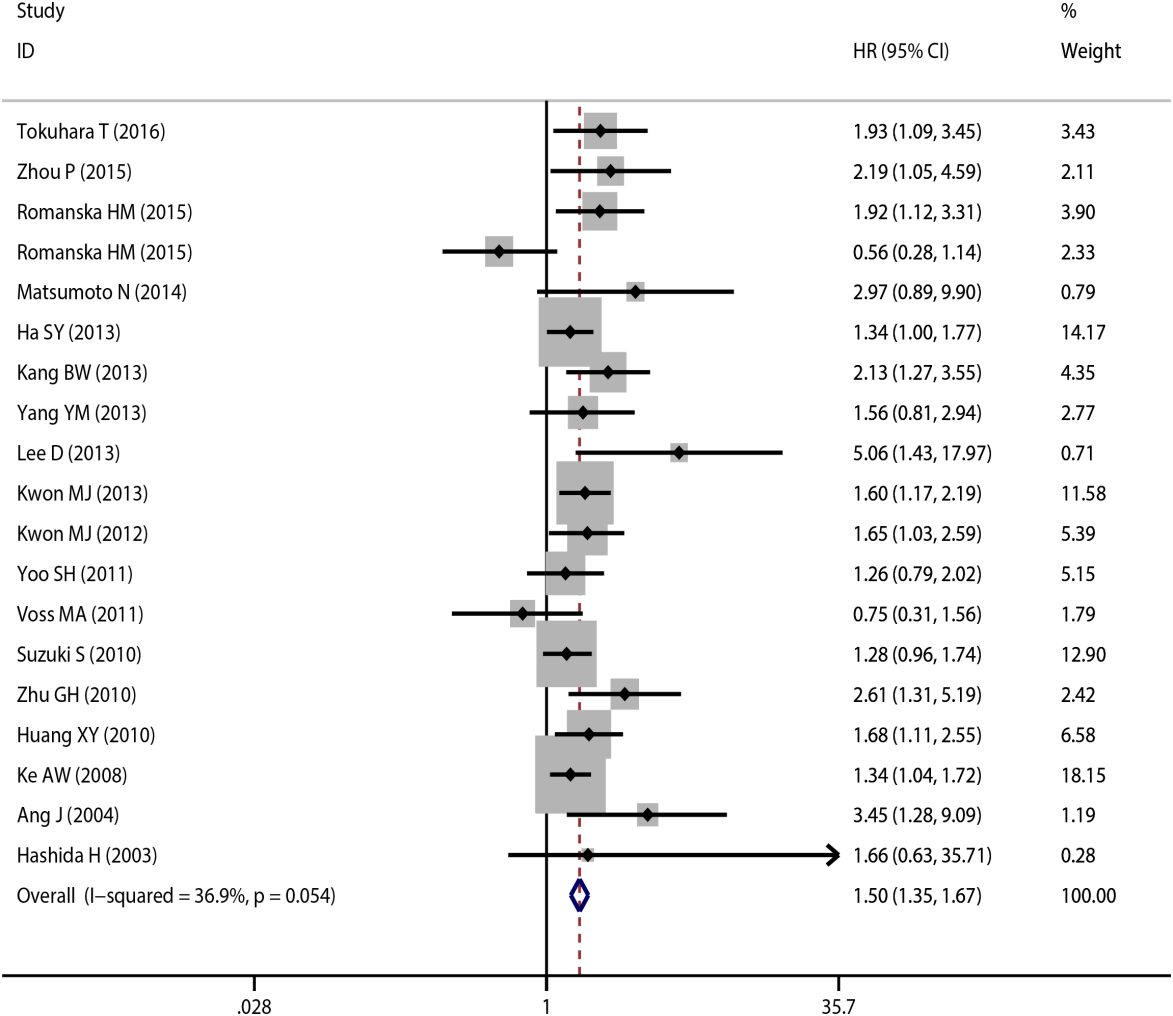
The correlation between CD151 expression and overall survival in solid tumor

**Figure 3:**
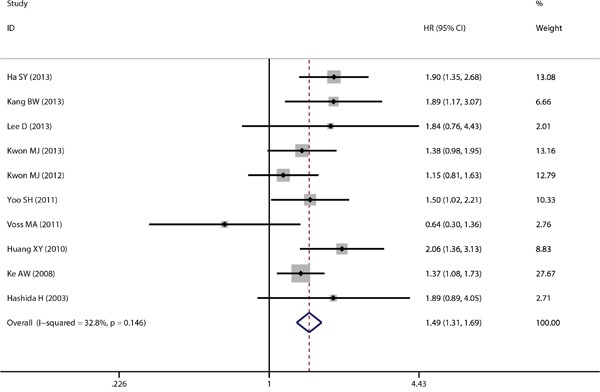
The correlation between CD151 expression and disease-free survival in solid tumor

As described, this meta-analysis was based on two outcome endpoints: OS and DFS. 19 studies were included in the current meta-analysis of OS. A fixed-effects model was utilized to calculate the pooled hazard ratio (HR) along with the 95% confidence interval (CI). Heterogeneity test reported a P value of 0.054 and an *I*^2^ value of 36.9%. The results indicated that CD151 overexpression was significantly associated with cancer patients’ poor OS (pooled HR =1.498, 95% CI = 1.346-1.667, *P*<0.001) (Figure 2). 10 studies were included in this current meta-analysis of DFS. Due to the fact that the heterogeneity test reported a P value of 0.146 and an *I*^2^ value of 32.8%, a fixed-effects model was again applied. The results demonstrated again a significant association between CD151 expression and DFS (pooled HR = 1.488, 95% CI = 1.314-1.685, *P*<0.001) (Figure 3). Subgroup study was then performed, the results indicated that the associations between CD151 overexpression and patients’ poor OS and patients poor DFS were also significant in Mongoloid patients (OS: pooled HR =1.511, 95% CI =1.348-1.693, *P*<0.001; DFS: pooled HR =1.524, 95% CI =1.343-1.729, *P*<0.001), as well in Caucasian (OS: pooled HR =1.400, 95% CI 1.018–1.924, *P*=0.038). The significant association was also detected between CD151 overexpression and poor OS in patients with breast cancer (OS: pooled HR = 1.399, 95% CI =1.022-1.915, *P*=0.036) and gastric cancer (OS: pooled HR = 1.498, 95% CI =1.188-1.890, *P*=0.001).

### Analysis of possible publication bias and sensitivity analysis

**Figure 4:**
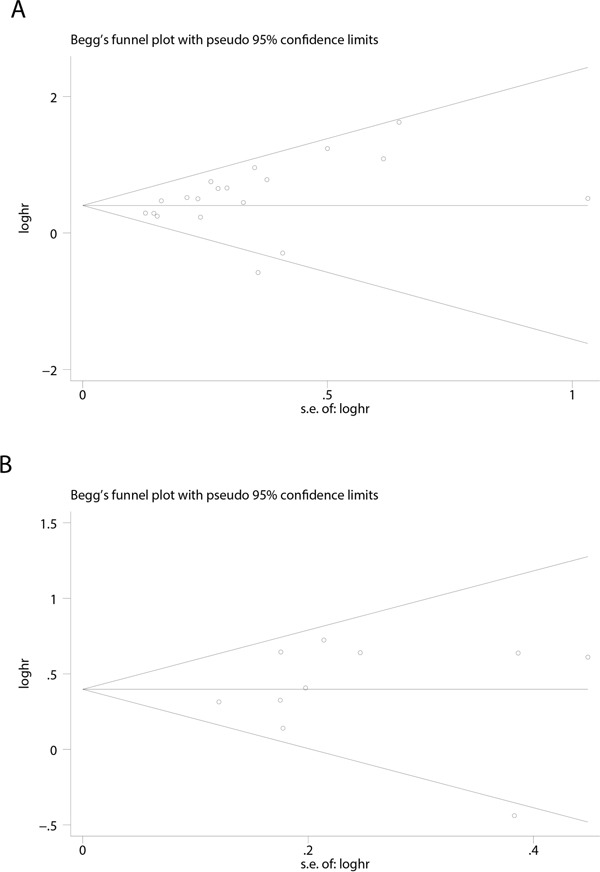
Begg's funnel plots for the studies involved in the meta-analysis of CD151 expression and the prognosis of patients with solid tumors **A**. Overall survival. **B**. disease-free survival(DFS). Abbreviations: loghr, logarithm of hazard ratios; s.e., standard error.

**Figure 5:**
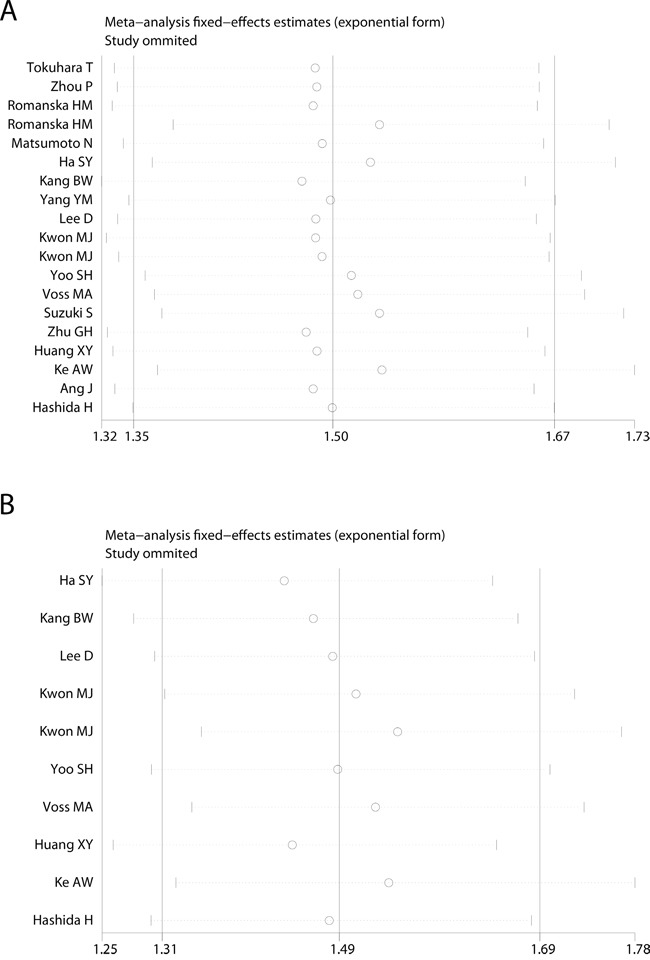
Sensitivity analysis of the meta-analysis **A**. Overall survival(OS). **B**. disease-free survival(DFS).

In this study, we utilized the Begg's funnel plot as well as the Egger's test to evaluate the possible publication bias. As demonstrated, the funnel plots shapes for the OS and DFS had no obvious heterogeneity (Figure 4), and Egger's tests revealed only slight publication bias concerning OS (P=0.097), but not DFS (P=0.839). Therefore, we performed trim and fill method to make pooled HR more reliable, and the pooled HR *P* value was also less than 0.01 (Figure not shown). Next, we performed the sensitivity analysis to determine the robustness of the above results. Not a single study was identified to dominate the current meta-analysis, and deletion of each single study had no significant impact on the general conclusions (Figure 5).

## DISCUSSION

Tetraspanin CD151 is composed of various structure domains [4]. Site-directed mutagenesis studies have revealed that CD151 could be palmitoylated at multiple sites, which is vital for the assemble of tumor endothelial marker (TEM) network [28, 29]. For instance, CD151 interacts with α3β1 and α6β4 integrins via intracellular N-terminal and C-terminal cysteine palmitoylation [28]. Studies have demonstrated that integrin subunits α3 and α6 may directly associate with CD151 at QRD194-196 site [30].

As the first identified member of the tetraspanin family, CD151 was shown to participate in tumor cell behaviors, including cell growth, proliferation, motility and invasiveness, possibly via interacting with a number of different proteins. CD151 silence could decrease above cancer cell behaviors [31–36]. Existing evidences have also shown that CD151 expression is also important for tumor neoangiogenesis and epithelial-tomesenchymal transition [35, 37].

In addition, clinical studies had investigated the potential prognostic value of CD151. Most of these studies, however, include only limited number of patients, and the results are inconclusive. CD151 overexpression often predicts unfavorable outcome in many cancer, such as gastric [11, 14, 24], prostate [10] and non-small cell lung cancer [18, 27]. On the other hand, it is a favorable prognostic indicator in invasive lobular breast cancer [15] and endometrial cancer [16]. To our knowledge, the present study is the first and most full-scale meta-analysis systemically exploring the possible prognostic role of CD151 up-regulation in solid malignancies.

We systematically evaluated survival data of 4, 270 solid tumor patients included in 19 different studies. Overall, these results clearly show that high CD151 expression could be a poor prognostic factor of various solid tumors, with both results of poor OS (pooled HR =1.498, 95% CI = 1.346-1.667, *P*<0.001) and poor DFS (pooled HR = 1.488, 95% CI = 1.314-1.685, *P*<0.001). Similarly, further subgroup analysis demonstrated the associations between CD151 overexpression and poor OS and DFS were significant within Mongoloid patients, as well poor OS in Caucasian. The results showed that there was no difference OS or DFS HR among different descent populations. When data was stratified according to cancer type, associations between CD151 overexpression and poor OS were also significant in breast cancer and gastric cancer. Together, our quantitative results strongly supported the current mainstream viewpoint that an undesirable impact of CD151 redundancy was correlated with not only the overall survival, but also disease-free survival. Additionally, Several important implications in this meta-analysis were displayed. High CD151 expression is very likely a general cancer's poor prognostic marker. We included a total of twelve different cancer types [10–27]. The pooled results showed that high CD151 expression was associated with patients’ poor OS and DFS. These conclusion could possibly be extended to all the solid tumors. Finally, it foreshadow the potential of CD151 developing a valuable therapeutic target and prognostic biomarker for solid tumor.

In conclusion, the significant association between CD151 over-expression and cancer patients’ poor survival was clearly demonstrated in the present meta-analysis. These results indicate that CD151 could be a potential prognostic biomarker and a promising therapeutic oncotarget for solid tumors.

## MATERIALS AND METHODS

### Publication search

This present meta-analysis was performed with the guidelines from Preferred Reporting Items for Systematics Reviews and Meta-Analyses [38]. Databases including PubMed, EMBASE and Web of Science were searched from their incipiency to June, 2016 using the search terms: ‘CD151’, ‘Tetraspanin’ and ‘cancer or tumor or neoplasm or carcinoma’ and ‘prognosis or prognostic or survival or predict or outcome or alive’ and the following limits: Human, article in English. All potentially eligible studies were retrieved. The bibliographies in these studies were also carefully scanned to identify other possible eligible studies and extra studies. At a situation when multiple studies of the same patient population were identified, the published report with the largest sample size was included.

### Inclusion criteria

To be eligible for selection of this meta-analysis, studies: (a) should test CD151 expression for prognostic value in cancer; (b) CD151 expression was tested by immunohistochemistry (IHC) or quantitative real-time polymerase chain reaction (qRT-PCR); (c) should have hazard ratios (HRs) with 95% confidence intervals (CIs), or should enable calculation of these statistics from the data presented; (d) should classify CD151 expression as “high” and “low” or “positive” and “negative”; (e) should be written in English.

### Exclusion criteria

Exclusion criteria were: (a) literatures such as letters, editorials, abstracts, reviews, case reports and expert opinions were excluded; (b) experiments were performed *in vitro* or *in vivo*, but were not associated with patients; (c) articles showing on HRs with 95% CI of OS, or the Kaplan-Meier curves which had inadequate survival data for further analysis; (d) The duration of the follow-up study was less than three years.

### Data extraction

Two outcome endpoints were analyzed: overall survival (OS) and disease-free survival (DFS). Parameters of these literatures were extracted from each single paper, including the first author's surname, publication year, country origin, number of total patients, antibodies utilized, types of measurements, score for CD151 assessment, CD151's cut-off values, as well as OS and DFS. The main features of these selected studies were summarized in Table 1.

**Table 1:** Characteristics of studies included in the meta-analysis

author	year	country	case	disease	antibody	method	cutoff value	outcome	NOS
								endpoints	score
Tokuhara T [27]	2016	Japan	145	non-small cell lung cancer	anti-CD151 monoclonal antibody SFA1.2B4	ICH	>50% of tumor cells stained	OS	7
Zhou P [26]	2015	USA	88	glioblastoma	anti-CD151 monoclonal antibodies (5C11)	ICH	≥15%cells positive	OS	7
Romanska HM(a) [15]	2015	Poland	182	invasive ductal breast carcinoma	mouse anti-human; 1 : 100; Novocastra, Newcastle upon Tyne, UK	ICH	scores of 2+ and 3+	OS	8
Romanska HM(b) [15]	2015	Poland	117	invasive lobular breast cancer	mouse anti-human; 1 : 101; Novocastra, Newcastle upon Tyne, UK	ICH	scores of 2+ and 3+	OS	8
Matsumoto N [20]	2014	Japan	45	gallbladder carcinoma	mouse monoclonal antibody against CD151 (ab33315; Abcam Inc, Cambridge, UK)	ICH	H score≥100 (range of 0-900)	OS	7
Ha SY [11]	2013	Korea	491	gastric carcinomas	mouse monoclonal anti-CD151 (NCL-CD151, 1:300 dilutio, Novocastra, Newcastle upon Tyne, UK)	ICH	score of 2+or 3+	OS, DFS	7
Kang BW [14]	2013	Korea	159	gastric cancer	mouse monoclonal anti-CD151 (NCL-CD151; 1:300 dilution, Novocastra, Newcastle upon Tyne, UK)	ICH	grade 2 and 3	OS, DFS	7
Yang YM [24]	2013	China	76	gastric cancer	Mouse anti-human CD151(11G5a, 1:200; Serotec, UK)	ICH	>50% of the tumor section	OS	9
Lee D [19]	2013	Korea	36	glioblastoma	mouse monoclonal anti-human CD151 (NCL-CD151, 1:300 dilution; Novocastra, Newcastle-upon-Tyne, UK)	ICH	grades 2 and 3	OS, DFS	6
Kwon MJ [18]	2013	Korea	380	non-small cell lung cancer	monoclonal mouse anti-human CD151 antibody(1:100 dilution, RLM30, Novocastra, Newcastle uponTyne, UK)	ICH	scores of 2+ and 3+	OS, DFS	8
Kwon MJ [25]	2012	Korea	886	breast cancer	monoclonal mouse anti-human CD151 antibody(1:100 dilution, RLM31, Novocastra, Newcastle uponTyne, UK)	ICH	score of 3+	OS, DFS	8
Yoo SH [22]	2011	Korea	489	clear cell renal cell carcinoma	Monoclonal mouse anti-human CD151 antibody (1:100, Novocastra, Newcastle upon Tyne, UK)	ICH	diffuse moderate or diffuse strong	OS, DFS	8
Voss MA [16]	2011	UK	131	endometrial cancer	Mouse anti-CD151 monoclonal antibody (mAb) (NCL-CD151, 1:50, Novocastra, Newcastle, Upon Tyne, UK)	ICH	H score≥100 (range of 0-300)	OS, DFS	8
Suzuki S [21]	2010	Japan	138	esophageal squamous cell carcinoma	mouse anti-CD151 monoclonal antibody (RLM30; 1:50, Novocastra, Newcastle, UK)	ICH	10% of tumor cells	OS	6
Zhu GH [23]	2010	China	71	Pancreatic cancer	mouse anti-human CD151 monoclonal antibody (sc-80715, 1:100 dilution, Santa Cruz Biotechnology, Santa Cruz, CA, USA)	ICH	3-7points	OS	8
Huang XY [13]	2010	China	140	intrahepatic cholangiocarcinoma	Monoclonal mouse anti-human CD151 (11G5a, 1:199; Serotec, UK)	ICH	>median	OS, DFS	8
Ke AW [17]	2008	China	520	hepatocellular carcinoma	Monoclonal mouse anti-human CD151 (11G5a, 1:200; Serotec, UK)	ICH	>50% of tumor section	OS, DFS	8
Ang J [10]	2004	Australia	30	prostate cancer	monoclonal mouse anti-human CD151 antibody (11B1, purified immunoglobulin IgG2a, 4 Ag/mL working concentration; ref. 10).	ICH	>the third quartile (17.52)	OS	8
Hashida H [12]	2003	Japan	146	colon cancer	anti-CD151 MAb SFA1.2B4	ICH	score≥120 (range of 0-300)	OS, DFS	8

ICH : Immunohistochemistry; NOS: Newcastle-Ottawa Scale;

There were two parts of data (a and b) in the study Romanska HM [15].

Cancer-specific survival, disease-specific survival and five-year OS were combined into OS. On the other hand, cumulative recurrence and progression-free survival were combined into DFS. OS was expressed as time (in months) from cancer diagnosis to death. To assess prognostic value of CD151 expression, multivariate hazard ratio (HR) was extracted. For the articles in which prognosis was plotted only as the Kaplan-Meier curves, the Engauge Digitizer V4.1 was then applied to extract survival data, and the Tierney's was utilized to calculate the HRs and 95% CIs [39].

### Statistical analysis

Pooled HRs and 95% CIs for two outcome endpoints (OS and DFS) were calculated. Statistical heterogeneity was assessed through the Chisquare test and *I*-square test, which was checked through the Q test, and a P value >0.10 indicated a lack of heterogeneity. We also quantified the effect of heterogeneity via *I*^2^ =100%×(Q - df)/Q. *I*^2^ values of <25% could be considered “low”, values of about 50% could be considered “moderate”, and values of over 75% could be considered “high” [40]. According to the absence or presence of heterogeneity, random-effects model or fixed-effects model was applied to merge the RR, respectively. Without statistical heterogeneity, a fixed-effects model was employed to calculate the pooled HRs, otherwise the random-effects model was performed [41]. Funnel plots and the Egger's test were utilized to determine the possible publication bias [26]. Sensitivity analysis was also tested. Statistical analyses were performed via the Stata 14.0 (StataCorp, College Station, TX). *P* values for all comparisons were two-tailed.
